# Diffuse Alveolar Hemorrhage: A Rare Life-Threatening Cause of Massive Hemoptysis

**DOI:** 10.7759/cureus.64347

**Published:** 2024-07-11

**Authors:** Amy E Hunt, Jessica Sop, Jarrod Kahre

**Affiliations:** 1 Emergency Medicine, Charleston Area Medical Center, Charleston, USA

**Keywords:** panton-valentine leucocidin, influenza b, methicillin-sensitive s. aureus (mssa), massive hemoptysis, diffuse alveolar hemorrhage

## Abstract

We present a case report of diffuse alveolar hemorrhage (DAH), which presented with massive hemoptysis and impending airway compromise. A previously healthy 33-year-old female presented to the emergency department with dyspnea, chest pain, and massive hemoptysis. Due to impending respiratory failure, the patient was placed on mechanical ventilation and a bronchoscopy revealed a diagnosis of DAH. Throughout the hospital course, the patient received antibiotics, steroids, fresh frozen plasma (FFP), cryoprecipitate, tranexamic acid (TXA), and multiple blood transfusions. The patient was subsequently placed on extracorporeal membrane oxygenation (ECMO), but despite these life-saving measures, the patient died less than 48 hours after her initial presentation. This case serves as a harrowing reminder of DAH’s destructive capabilities and the importance of rapid, aggressive management.

## Introduction

Diffuse alveolar hemorrhage (DAH) is a rare clinical syndrome, with an incidence rate of 2%, and is a potentially life-threatening condition that requires early diagnosis and rapid intervention. This condition is characterized by cough, hemoptysis, diffuse pulmonary infiltrates, anemia, and hypoxemic respiratory failure. Symptoms may appear acutely or over a period of time [1-3]. In-hospital mortality associated with DAH generally ranges from 20% to 50% but has been reported to be as high as 100% in some studies [2,4]. Various etiologies can cause DAH, including systemic vasculitis, rheumatic diseases, and drug toxicities. Respiratory infections can also lead to DAH pathology with influenza A (H1N1), malaria, leptospirosis, dengue, and* Staphylococcus aureus* infections being the most common in immunocompetent patients [3,5]. Panton-Valentine leucocidin (PVL) is a cytotoxin believed to be present in less than 5% of *S. aureus* strains and can cause tissue necrosis, necrotizing pneumonia, and predispose individuals toward DAH [6]. Our case illustrates a young patient who was diagnosed with *S. aureus* pneumonia and ultimately DAH and highlights the devastating potential of DAH. 

## Case presentation

A 33-year-old white female presented to the emergency department with chest pain, dyspnea, and hemoptysis, which began just hours prior to her arrival. She indicated she had been experiencing upper respiratory symptoms over the past three to four days and had recently tested positive for influenza B. Of note, the patient stated that she had an elective bilateral breast augmentation surgery performed three weeks ago and she had traveled from out of state one day prior to the current presentation. She had a history of Graves' disease and took only desiccated thyroid. There was no reported history of tobacco, alcohol, or illicit drug use. 

**Figure 1:**
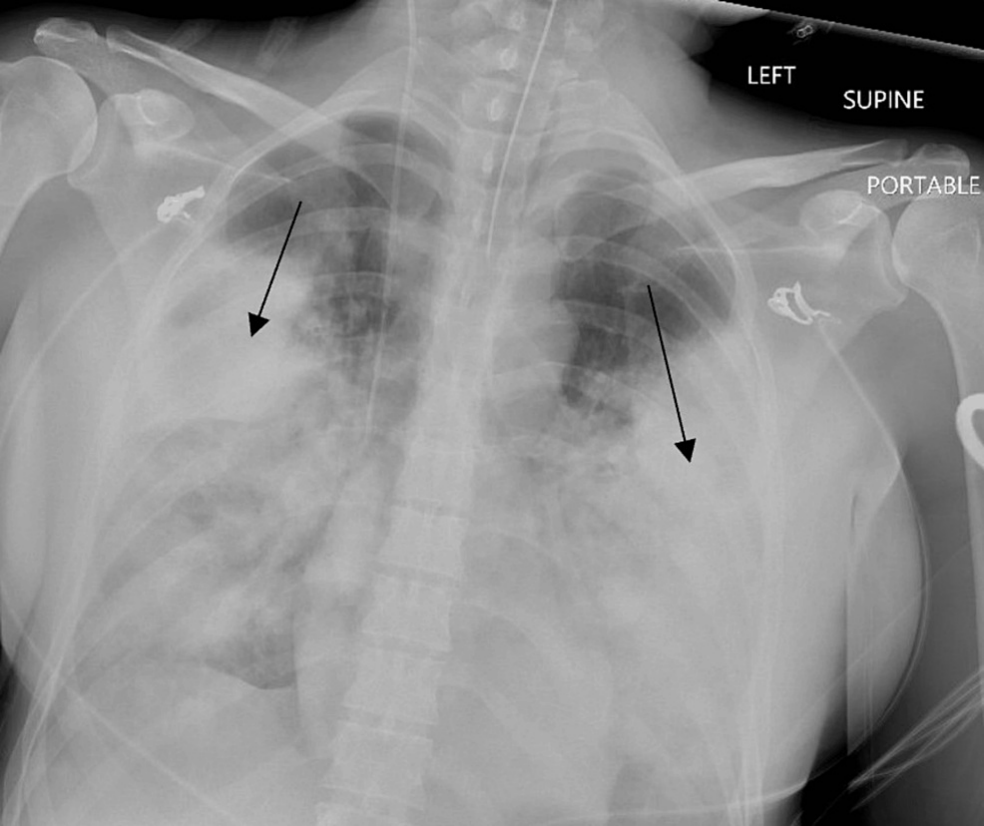
Chest X-ray obtained in the emergency department demonstrates dense infiltrates within the mid-lung fields bilaterally. A right internal jugular central line terminates in the mid-superior vena cava. The endotracheal tube remains in place. The heart is within normal limits for size. No effusion or pneumothorax is seen.

Upon arrival at the emergency department, the patient was afebrile with a temperature of 36.5°C, heart rate of 170 beats per minute, blood pressure of 106/55 mmHg, respiratory rate of 30 breaths per minute, and oxygenation of 64% in room air. On physical examination, the patient was dyspneic and appeared cyanotic; she was also having frequent hemoptysis at the bedside and was in obvious respiratory distress. Due to impending airway compromise, the patient was endotracheally intubated shortly after arrival. During intubation, a brisk stream of bright red blood was visualized and appeared to be originating distal to the vocal cords raising further suspicion that the bleed was of pulmonary rather than gastrointestinal origin. Chest X-ray confirmed appropriate endotracheal tube and central line placement along with significant bilateral airspace opacities (Figure 1).

**Figure 2:**
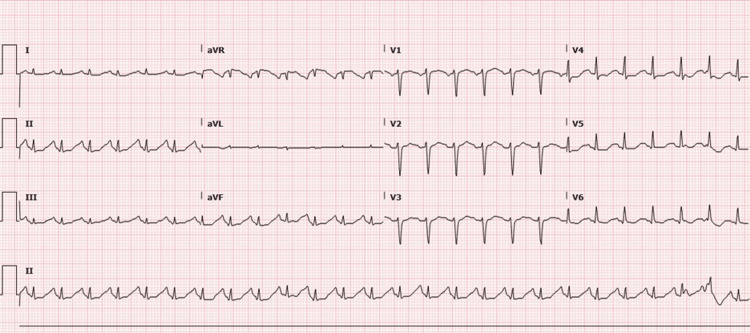
Initial EKG reveals sinus tachycardia with a heart rate of 153 beats per minute. Low voltage in extremity and precordial leads. EKG: electrocardiogram

**Figure 3:**
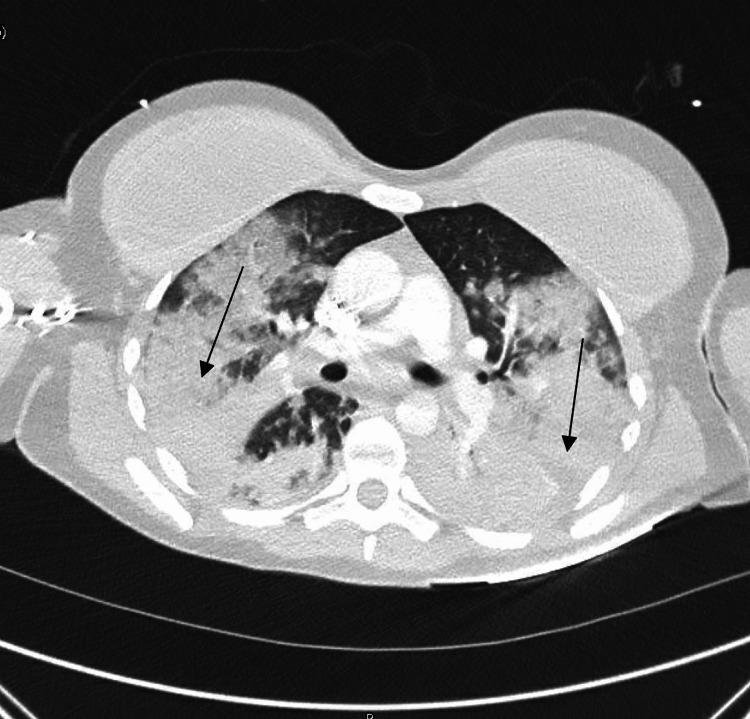
CTA of the chest reveals extensive bilateral consolidation, which may represent pneumonia, pulmonary hemorrhage, or acute respiratory distress syndrome. No evidence of pulmonary embolism. CTA, computed tomography angiography

Laboratory evaluation revealed respiratory acidosis and profound leukopenia (Table 1). An electrocardiogram (EKG) revealed sinus tachycardia (Figure 2). A contrast-enhanced computed tomography angiography (CTA) was completed and demonstrated extensive bilateral consolidations and no evidence of a pulmonary embolism (Figure 3). During her course in the emergency department, the patient required maximum support on mechanical ventilation with frequent in-line suctioning to maintain oxygenation. An emergent bronchoscopy revealed diffuse blood in the airways, which was only briefly controlled with direct cold saline lavage. Two liters of blood were suctioned from the patient’s lungs over the span of one hour. The patient was given three liters of intravenous normal saline fluid, linezolid, and two units of uncrossmatched red blood cells, and was started on vasopressors for continued hypotension. The patient was subsequently admitted to the intensive care unit (ICU).

**Table 1:** Initial laboratory evaluation from the emergency department. pH: blood pH level; pCO_2_: partial pressure of carbon dioxide; pO_2_: partial pressure of oxygen; HC0_3_: bicarbonate; O_2_ saturation: oxygen saturation; Hgb: hemoglobin; C0_2_: carbon dioxide; BUN: blood urea nitrogen; GFR: glomerular filtration rate; aPTT: activated partial thromboplastin clotting time; PT: prothrombin time; INR: international normalized ratio; HS: high sensitivity

Laboratory Test	Value	Reference Range
pH	7.09	7.35-7.45
pCO_2_	77 mmHg	32-45 mmHg
pO_2_	86 mmHg	83-108 mmHg
HCO_3_-	22 mmol/L	NA
O_2_ Saturation	91%	95-97%
White Cell Count	1.0x10^3^/mcL	4.8-10.8x10^3^/mcL
Red Cell Count	5.42x10^6^/mcL	4.2-5.4x10^6^/mcL
Hemoglobin	16.3 g/dL	12-15 g/dL
Hematocrit	47.9%	36-47%
Mean Cell Volume	88.5 fL	80-100 fL
Mean Cell Hgb	30.2 pg	27-32 pg
Platelet Count	107x10^3^/mcL	140-450x10^3^/mcL
Segmented Neutrophil	4%	50-70%
Band Neutrophil	10%	0-8%
Lymphocyte	71%	20-35%
Monocyte	5%	0-13%
Eosinophil	1%	0-4%
Sodium	139 mmol/L	136-145 mmol/L
Potassium	3.2 mmol/L	3.5-5.1 mmol/L
Chloride	116 mmol/L	98-107 mmol/L
CO_2_, Whole Blood	21 mmol/L	21-32 mmol/L
Glucose	162 mg/dL	74-106 mg/dL
BUN	10 mg/dL	7-25 mg/dL
Creatinine	1.4 mg/dL	0.6-1.2 mg/dL
Estimated GFR	53 mL/min/1.73 m^2^	>/=60 mL/min/1.73 m^2^
Lactic Acid	8.8 mmol/L	0.5-2.0 mmol/L
aPTT	33.9 seconds	25.1-36.5 seconds
PT	14.1 seconds	9.4-12.5 seconds
INR	1.25	NA
Troponin-I HS	3 pg/mL	15 pg/mL

In the ICU, the patient required escalating doses of multiple vasopressors. Despite maximum support on mechanical ventilation, frequent suctioning, and a repeat bronchoscopy, the patient’s oxygenation worsened. Twelve hours after the initial presentation, she was placed on venovenous extracorporeal membrane oxygenation (ECMO) and later venoarterial ECMO. She developed an oliguric acute kidney injury and was placed on continuous renal replacement therapy (CRRT). Immunologic evaluation revealed negative anti-nuclear antibody (ANA) and antineutrophil cytoplasmic antibody (ANCA) levels. Phospholipid antibodies, proteinase antibodies, and human immunodeficiency virus (HIV) screen were also negative. Her viral respiratory panel returned positive for influenza B, and the pneumonia panel PCR obtained via bronchoalveolar lavage (BAL) was positive for methicillin-sensitive *Staphylococcus aureus* (MSSA), *Haemophilus influenzae*, and influenza B. Both blood and respiratory cultures were positive for MSSA. Fungal culture, acid-fast bacilli culture, legionella antigen, and pneumocystis smear were negative.

During her course in the ICU, the patient received antibiotics, high-dose steroids, fresh frozen plasma (FFP), cryoprecipitate, platelet transfusions, tranexamic acid (TXA), as well as numerous blood transfusions. The patient’s vasopressor support included the administration of norepinephrine, epinephrine, vasopressin, phenylephrine, and angiotensin II. The patient subsequently displayed mottling of her upper and lower extremities, whereby an arterial ultrasound demonstrated no arterial flow to her bilateral radial and ulnar arteries, an occluded distal right anterior and posterior tibial artery, and a complete occlusion of her left posterior and anterior tibial arteries. Despite maximal medical resuscitation, the patient died within 48 hours of her initial presentation. An autopsy revealed pulmonary findings consistent with DAH, which was listed as the official cause of death.

## Discussion

DAH is a serious, potentially life-threatening syndrome that mandates aggressive evaluation and intervention. DAH can also be referred to as intrapulmonary hemorrhage, diffuse pulmonary hemorrhage, pulmonary alveolar hemorrhage, pulmonary capillary hemorrhage, alveolar bleeding, or microvascular pulmonary hemorrhage [7]. Because hemoptysis is absent in up to one-third of cases and other symptomatology and radiographic findings are non-specific, early bronchoscopy with BAL is often required to diagnose DAH. The most common histological finding identified in DAH by biopsy is pulmonary capillaritis; however, this does not establish the specific cause necessitating further ancillary studies [1,7,8].

Consequential to the high mortality rate associated with DAH, it is imperative to identify the etiology behind DAH development to direct appropriate treatment. The causes of DAH have been differentiated into several categories. One classification identifies immune-associated, congestive heart failure (CHF)-associated, miscellaneous, and idiopathic, which can alternatively be identified as immune-mediated and non-immune-mediated. Another more simplistic categorization is infectious versus noninfectious [3,9]. There are a multitude of conditions that cause DAH; however, there is no prospective study that identifies the most common cause. Systemic vasculitis is frequently associated with DAH and one report of 34 DAH cases found that Wegener’s granulomatosis, often referred to as granulomatosis with polyangiitis, accounted for the highest percentage of DAH cases [1,7,10].

More rarely, pulmonary infections are the cause of DAH. Cytomegalovirus, adenovirus, aspergillosis, legionella, mycoplasma, and strongyloides are the more frequent causes of DAH in immunocompromised patients, while influenza A (H1N1), malaria, leptospirosis, dengue, and *S. aureus* infections are more common causes in immunocompetent hosts [3,5]. The patient in this case was diagnosed with influenza B and had positive blood and sputum cultures for methicillin-sensitive *S*. *aureus*. There have been numerous cases of severe, often fatal, community-acquired Panton-Valentine leucocidin (PVL) secreting *S*. *aureus* pneumonia reported in the literature. It often occurs in young, immunocompetent patients who have an influenza-like prodromal illness [11,12]. Less than 5% of *S. aureus* strains produce PVL, and its cytotoxic effects destroy neutrophils and cause tissue necrosis [6]. PVL-producing *S. aureus* pneumonia is necrotizing and post-mortem evaluations have often revealed significant airway necrosis and lung hemorrhage [11]. It is theorized that preceding influenza infection causes lung epithelium to attract neutrophils, which are subsequently killed after co-infection with *S. aureus* by PVL. This then causes a protease release resulting in significant airway epithelial destruction [13]. When Vardakas et al. compared community-acquired pneumonia (CAP) due to PVL-secreting MRSA and MSSA, there was no significant difference in mortality. They reported that features related to MRSA CAP were gastrointestinal tract symptoms and unilobar infiltrates, while respiratory distress syndrome, airway hemorrhage, and multilobar infiltrates were associated with MSSA CAP [14]. Airway bleeding, erythroderma, and leukopenia are factors related to mortality in community-acquired PVL-positive *S. aureus* pneumonia [15]. Although the *S. aureus* infecting the patient, in this case, was not tested for PVL, it is suspected that it would have been positive given the similarities in her presentation and course to other reported cases of fatal community-acquired PVL-secreting *S. aureus* pneumonia. The patient was young, immunocompetent, and had a concomitant influenza infection. She also had leukopenia and airway bleeding upon presentation, which are conditions associated with increased mortality in CAP due to PVL-secreting *S. aureus* pneumonia. Furthermore, her evaluation for the more common cause of DAH, which is an autoimmune disease, was negative.

As aforementioned, it is critical to identify the cause of DAH to render appropriate therapy. Treatment of DAH associated with autoimmune disorders and vasculitides consists of corticosteroids, immunosuppressive agents, and intravenous immunoglobulin (IVIG). Plasmapheresis may be implemented when titers of pathogenic immunoglobulins are high. In instances of significant coagulopathy, platelets, vitamin K, and cryoprecipitate should be given while hemostatic agents such as TXA, epsilon aminocaproic acid (EACA), thrombin, and factor VIIIa should be considered. Diuresis should be initiated in CHF-related DAH [7,9]. DAH caused by infection is treated with broad-spectrum antibiotics, antivirals, and IVIG depending on the causative pathogen [16,17]. Acute respiratory distress syndrome leading to respiratory failure necessitates mechanical ventilation and in cases unresponsive to mechanical ventilation, the use of venovenous and venoarterial ECMO has improved outcomes [18,19]. Considering the hospital course of the patient in this case, adjuvant IVIG could have been considered as one study has shown that it offsets the cytotoxic effects of PVL in vitro [17].

## Conclusions

This case serves as an important reminder of DAH’s devastating potential illustrating the rapid deterioration in an otherwise healthy, immunocompetent individual. DAH should be high on the differential for patients who present with sudden-onset massive hemoptysis. It is imperative physicians identify the cause of DAH in a timely manner to render appropriate therapy and help curb the in-hospital mortality rate of this disease process. In cases when the infectious etiology of DAH is suspected, all therapeutic options should be considered, including those that may not be commonly utilized in septic shock such as antivirals and IVIG. DAH is so volatile that even early diagnosis and aggressive management may not be sufficient to prevent mortality as evidenced by this case report.
